# Comparison of autologous venous grafts and three types of extracellular matrix grafts in Peyronie’s disease surgery

**DOI:** 10.55730/1300-0144.5865

**Published:** 2024-05-23

**Authors:** Türker SOYDAŞ, Selman ÜNAL, Halil UZUNDAL, Merve KAŞIKCI, Musab Ali KUTLUHAN, Emrah OKULU, Önder KAYIGİL

**Affiliations:** 1 Department of Urology, Ankara Etlik City Hospital, Ankara, Turkiye; 2 Department of Urology, Ürgüp State Hospital, Nevşehir, Turkiye; 3 Department of Urology, Ankara Mamak State Hospital, Ankara, Turkiye; 4 Department of Biostatistics, Faculty of Medicine, Hacettepe University, Ankara, Turkiye; 5 Department of Urology, Faculty of Medicine, Yıldırım Beyazıt University, Ankara, Turkiye

**Keywords:** Peyronie’s disease, Peyronie, grafting, grafting procedures

## Abstract

**Background/aim:**

Peyronie’s disease (PD) is known as a wound-healing disorder for which surgery remains the gold-standard treatment, but studies comparing graft materials are limited in the literature. The aim of this study was to evaluate the mid- and long-term results of patients who underwent surgery for PD with grafting procedures performed by a single experienced surgeon according to graft materials.

**Materials and methods:**

Patients who underwent corporoplasty between 2014 and 2020 with grafting procedures performed by a single experienced surgeon were retrospectively reviewed. A total of 115 patients were divided into 4 groups according to the grafting material used: autologous saphenous venous grafts, Group 1 (n = 36); porcine pericardial extracellular matrix grafts (EMGs; XenoGuard, MBP Medical Biomaterial Products GmbH, Neustadt-Glewe, Germany), Group 2 (n = 40); porcine intestinal submucosal EMGs (BioDesign, Cook Medical, Bloomington, IN, USA), Group 3 (n = 36); and bovine pericardial EMGs (Tutopatch, Tutogen Medical, Inc., Alachua, FL, USA), Group 4 (n = 43).

**Results:**

The mean operation time for Group 1 was longer than that of the other groups (p < 0.001). When comparing the groups in pairs, it was observed that the duration of postoperative loss of sensation (LOS) was significantly shorter in Group 3 (12.3 ± 5.3 days) and Group 4 (15.1 ± 3.1 days) (p < 0.05). There was a statistically significant difference between Groups 1 and 4 in penile length loss when the groups were compared in pairs (p = 0.017). There was a statistically significant difference between patients with penile curvatures of 0° to 59° and patients with curvatures of ≥60° in terms of duration of postoperative LOS (14.4 ± 5 vs. 16.4 ± 5.8 days, respectively; p = 0.028) and penile length loss (2.6 ± 5 vs. 5.7 ± 6.8 mm, respectively; p = 0.002).

**Conclusion:**

The findings suggest that EMGs should be preferred to autologous venous grafts due to reduced postoperative erectile dysfunction, shorter operation time, and shorter recovery time for LOS.

## 1. Introduction

Peyronie’s disease (PD) is known as a wound-healing disorder characterized by penile pain, penile curvature, and sexual dysfunction. Although the pathophysiology of PD remains elusive, it is attributed to repeated minor trauma of the erect penis and abnormal healing of the tunica albuginea [1,2]. Surgery remains the gold-standard treatment for PD-related deformity. In surgical treatment, plication techniques, grafting techniques, a combination of plication and grafting techniques, and imbrication techniques can be applied in the presence of cavernous sacculation [3].

There are many grafting materials, including autologous grafts, synthetic grafts, allografts, and xenografts. The most important disadvantage of autologous grafts is increased surgery time and harvesting complications. Synthetic grafts (Dacron or polytetrafluoroethylene) are no longer recommended due to increased risks of inflammation, fibrosis, and infection [4]. Allografts and xenografts have been used in surgery for PD, but there are limited studies in the literature evaluating mid- and long-term results or comparing multiple graft types. We previously published our experience with autologous venous grafts and one type of xenograft for the surgical correction of PD [5].

This study aimed to evaluate the mid- to long-term results of patients who underwent PD surgery using autologous saphenous venous grafts or one of three types of xenografts (porcine pericardial extracellular matrix grafts [EMGs], porcine intestinal submucosal EMGs, and bovine pericardial EMGs).

## 2. Materials and methods

**Figure 1:**
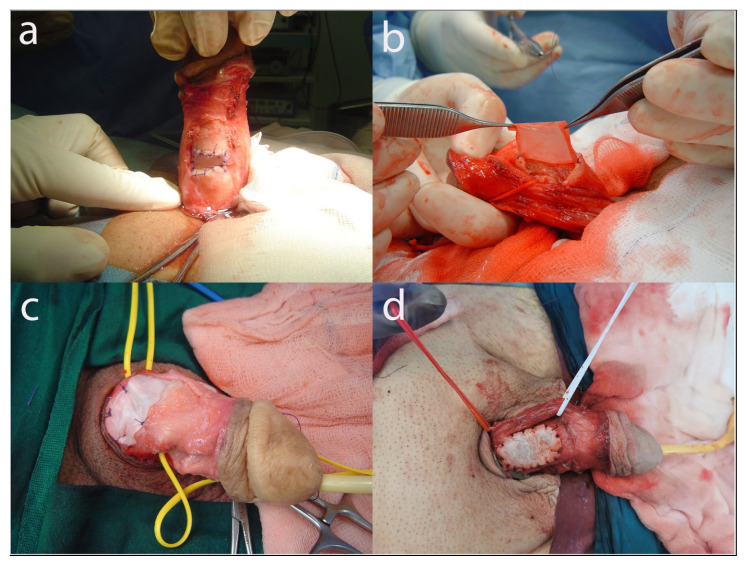
(a) Peyronie’s disease surgery with autologous saphenous vein graft, (b) Peyronie’s disease surgery with porcine pericardium EMG, (c) Peyronie’s disease surgery with small intestinal submucosal EMG, and (d) Peyronie’s disease surgery with bovine pericardial EMG. All photos are from Önder Kayıgil’s archive.

Patients who underwent corporoplasty with penile plication and grafting procedures by a single experienced surgeon between 2014 and 2020 were retrospectively evaluated. A total of 115 patients were included in the study and divided into 4 groups according to the graft material used: autologous saphenous venous grafts, Group 1 (n = 36, Figure 1a); porcine pericardial EMGs (XenoGuard, MBP Medical Biomaterial Products GmbH, Neustadt-Glewe, Germany), Group 2 (n = 40, Figure 1b); porcine intestinal submucosal EMGs (BioDesign, Cook Medical, Bloomington, IN, USA), Group 3 (n = 36, Figure 1c); and bovine pericardial EMGs (Tutopatch, Tutogen Medical, Inc., Alachua, FL, USA), Group 4 (n = 43, Figure 1d). Patients who underwent penile revascularization and penile prosthesis in the same session were excluded from the study to objectively evaluate postoperative erectile results. Patients who did not continue their postoperative follow-up were also excluded from the study. The study protocol was approved by the Yıldırım Beyazıt University Clinical Research Ethics Committee (Decision No: 26379996/129; Date: 16.12.2020).

### 2.1. Preoperative and intraoperative evaluations

Preoperative evaluations included the age and body mass index of patients, history of disease onset and duration, pain with or without erection, and comorbidities. Penile color duplex ultrasonography was preoperatively performed to evaluate erectile function. Preoperative and postoperative penile length measurements, preoperative and 3-month postoperative International Index of Erectile Function (IIEF-5) scores, penile curvature degree, penile curvature direction, presence of complex deformities (hinge or hourglass), presence of cavernous sacculation, type and size of graft used during surgery, whether or not urethral dissection was performed, postoperative complications (wound infection, severe edema, hypoesthesia, cavernous leak, hematoma, delay in ejaculation, anejaculation, painful palpable plication suture), penile length loss (PLL), and loss of sensation (LOS) were evaluated from patient files and electronic record systems. All patients had a history of stable disease for at least 6 months before surgery.

### 2.2. Surgical technique

Penile deformities were evaluated after penile degloving and artificial erection. Subsequently, the tunical tissue was reached by an incision made from the lateral of the bilateral urethra to Buck’s fascia, and then the neurovascular tissues were separated from the tunical tissue by applying sharp dissections. The maximal deformity was evaluated by artificial erection with saline infusion with a 21-gauge butterfly needle, and plication was performed with 0 or 1/0 Prolene sutures. The presence of residual curvature was then evaluated with artificial erection. Afterward, a plaque incision or partial excision was performed. From the maximal tension point of the plate, the tunical tissue was incised transversely with a Y-shaped incision at both ends up to the cavernous tissues, and then the calcified plaque was partially excised. Excision of the entire plaques was performed only for some patients with extremely calcified plaques. Tunical shaving was applied on the tunical tissue with a number 15 scalpel. The graft material was cut to the size of the defect after excision and sutured intermittently with 4/0 Vicryl sutures. We previously published a study demonstrating that performing plication before graft application reduces the size of the graft [3]. Buck’s fascia was repaired by intermittent suturing with 3/0 Vicryl sutures, followed by subcutaneous and skin suturing.

If cavernous sacculation was detected, the deformity was corrected by intermittent suturing with 1/0 Prolene sutures over the metal route placed on the tunical tissue, as described by Kayıgil et al. [3]. A Coban bandage was applied at the end of the operation.

### 2.3. Postoperative penile rehabilitation

Patients were evaluated in the 1st postoperative week, penile massage and stretching exercises were recommended in the postoperative 2nd week, and a vacuum device was recommended in the 4th postoperative week to be applied for 3 months by increasing the pressure until the pain tolerance point 3 times a day for periods of 5–10 min. From the 4th postoperative week, massage and stretching exercises were recommended to be performed by the sexual partner, if possible. From the 2nd postoperative week, 5 mg of PDE5 inhibitor once daily in the evening for 3 months was recommended. Sexual abstinence was recommended for 2 months postoperatively.

### 2.4. Statistical analysis

Statistical evaluation was performed using IBM SPSS Statistics 23.0 (IBM Corp, Armonk, NY, USA). Data were expressed as frequencies and percentages for categorical variables, medians for numerical variables, and interquartile range, minimum, maximum, mean, and standard deviation values. The difference between two independent groups was evaluated by the Mann–Whitney U test. The difference between more than two groups was evaluated with the Kruskal–Wallis test. When comparing more than two independent groups with at least one of the groups presenting a difference, the group or groups causing the difference were determined by multiple comparison tests (post hoc tests). Whether there was a difference between two dependent groups was assessed with the Wilcoxon test. The relationship between two numerical variables was evaluated with the Spearman correlation coefficient. The evaluation of covariance of categorical variables was analyzed using chi-square analysis. Values of p < 0.05 were considered statistically significant.

## 3. Results

The mean age of the patients was 52.4 ± 14.4, 53.7 ± 10.7, 57 ± 8.6, and 51.6 ± 11.7 years in Groups 1, 2, 3, and 4, respectively. The age of the patients ranged from 27 to 70 years. The groups were homogeneous in terms of hypertension, diabetes mellitus, coronary heart disease, trauma history, hyperlipidemia, and smoking history. The demographic features of patients are summarized in Table 1.

In preoperative evaluations, the groups were homogeneous in terms of IIEF-5 scores (Table 2). The mean IIEF-5 scores evaluated at the 6th postoperative month were 17.7 ± 4 for Group 1, 19.9 ± 4.5 for Group 2, 17.8 ± 3.1 for Group 3, and 19.4 ± 2.1 for Group 4 (p = 0.011). Preoperative and postoperative differences in IIEF-5 scores were statistically significant for Group 2 (p = 0.001) and Group 4 (p = 0.015). The mean graft size used in the operation was similar between groups.

The mean operation time was 134.8 ± 17.9 min for Group 1, 65.4 ± 6.9 min for Group 2, 63 ± 6.6 min for Group 3, and 62.9 ± 7.1 min for Group 4. The operation time for Group 1 was found to be statistically significantly longer compared to the other groups (p < 0.001, Table 2). Complex deformities were detected in two patients in Group 1, two patients in Group 2, and three patients in Group 4; no complex deformities were detected among the patients of Group 3.

Longitudinal imbrication suturing was applied for seven (19.4%) patients in Group 1, one (2.3%) patient in Group 2, two (5%) patients in Group 3, and one (3%) patient in Group 4. Intraoperative features of the patients are summarized in Table 3.

Wound infection, incision contracture, inguinal hypoesthesia in the 2nd postoperative week, anejaculation, painful plication suture, and palpable suture complications were not observed in any patients in the study population. Complications requiring secondary surgical operation were not observed. All encountered complications are listed in Table 4.

There was a statistically significant difference between groups in terms of postoperative duration of LOS (p < 0.001). When the groups were compared in pairs, the postoperative duration of LOS was statistically significantly shorter in Group 3 (12.3 ± 5.3 days) and statistically significantly longer in Group 1 (18.9 ± 4.8 days) (p < 0.05, Table 5).

A statistically significant difference was observed in mean PLL between Groups 1 and 4 when the groups were compared in pairs (5.8 ± 7.0 vs. 1.8 ± 4.1 mm, respectively; p = 0.017, Table 5). The mean PLL was 2.6 ± 5 mm in the group of patients with curvatures ranging from 0° to 59° and 5.7 ± 6.8 mm in the group of patients with curvatures of ≥60° (p = 0.002, Table 6).

When the relationship between penile curvature degrees and postoperative duration of LOS was evaluated, a statistically significant difference was found between patients with curvatures ranging from 0° to 59° and patients with curvatures of ≥60° in terms of postoperative duration of LOS (14.4 ± 5 vs. 16.4 ± 5.8 days, respectively; p = 0.028) and PLL (2.6 ± 5 vs. 5.7 ± 6.8, respectively; p = 0.002, Table 6).

## 4. Discussion

To date, the ideal graft material for the correction of tunica albuginea deformities has not been defined. In this respect, our study is important in terms of both comparing EMG types with autologous venous graft materials and being the first study in the literature to explore the use of three different types of ECM graft materials in surgery for PD.

EMGs tend to promote growth factors by accelerating tissue growth and revascularization [6]. When the groups were compared in pairs for postoperative LOS, the recovery time of the patients in Group 3 was statistically significantly shorter and that of the patients in Group 1 was statistically longer (p < 0.001). Although the recovery time in Group 2 was shorter than that in Group 4, the difference was not statistically significant. The EMGs may have been superior to each other or differences may have been caused by patient-related factors. However, results show that EMGs heal faster than autologous venous graft in terms of LOS. In a study by Sayedahmed et al. [7], transient LOS in the glans penis was noted in seven cases. Further studies are required to obtain clear evidence of faster tissue healing in groups treated with EMGs.

The duration of LOS was shorter in patients with curvatures ranging from 0° to 59° compared to patients with curvatures of ≥60° (14.4 ± 5 vs. 16.4 ± 5.8 days, respectively; p = 0.028). Since LOS according to curvature degree has not been previously evaluated in the literature, this finding needs to be supported by more studies.

The increase in IIEF-5 scores observed in this study after surgery may be due to the presence of deformities that caused difficulty in sexual intercourse before surgery and the feelings of embarrassment that may develop psychologically secondary to such deformities. The postoperative increase in IIEF-5 scores was statistically significant for Group 2 (p = 0.001) and Group 4 (p = 0.015). This could be attributed to the superiority of certain graft types over others, but the result should be supported by randomized prospective studies with larger patient groups. In our previously published study [5], the mean preoperative and postoperative IIEF-5 scores were 18 in the patient group treated with autologous venous grafts, while the preoperative IIEF-5 score was 20 and the postoperative IIEF score was 21 in the group treated with EMGs. In the study by Sayedahmed et al. [7], the mean IIEF-5 score was 16 preoperatively and 20 postoperatively. In that study, the group with the highest increase in IIEF-5 scores was the patient group with preoperative IIEF-5 scores of 12–16.

The risk of erectile dysfunction developing in patients undergoing grafting procedures for PD varies between 4% and 53.8% [8]. It is thought that the graft size and the risk of postoperative erectile dysfunction may be related, but there are not enough data in the literature for firm conclusions. The mean graft size used in the operations was 198.4 ± 160.5 mm^2^ for Group 1, 224.2 ± 182.2 mm^2^ for Group 2, 154.7 ± 90.4 mm^2^ for Group 3, and 156.2 ± 74 mm^2^ for Group 4. The patients in Group 2 had the highest mean graft size, and so the statistically significant increase in IIEF-5 scores in this group suggests that postoperative erectile dysfunction is not clearly related to graft size. Although the increase in IIEF-5 scores for patients in Group 2 and Group 4 was statistically significant, there were increases in the IIEF-5 scores of all groups and none of the patients had problems with axial rigidity during sexual intercourse in the postoperative period; this suggests that autologous grafts and EMGs mimic the tunical tissue very well and have good tissue integration.

When the results were evaluated in terms of mean PLL, a statistically significant difference was observed between Groups 1 and 4 (5.8 ± 7 mm vs. 1.8 ± 4.1 mm, respectively; p < 0.001). A mean PLL of 5 ± 6.6 mm was observed in Group 2 and a mean loss of 2.7 ± 4.9 mm occurred in Group 3. The fact that there were fewer patients in Group 4 with a high degree of curvature (≥60°) compared to the other groups may explain why PLL was lowest in this group. According to the review published by Rice et al. [8], penile shortening is observed in 4.9% to 40% of patients undergoing PD repair with grafts, penile length does not change in 44.2%–95%, and a penile length increase is observed in 5%–48.8%.

The mean follow-up period was 80.3 ± 27.8 weeks for Group 1, 39.3 ± 11.1 weeks for Group 2, 56.1 ± 14.9 weeks for Group 3, and 62.6 ± 10.9 weeks for Group 4. In the literature, the follow-up period of patients undergoing PD repair with grafts varies between 12 and 51 months [8]. The follow-up times in our study are similar to those in the literature.

This study has some limitations. First, the study was not designed as a prospective randomized controlled study. Second, we used the IIEF-5 for postoperative erectile function, but this questionnaire could lead to misleading results in these patient groups. The strength of our study is that all surgeries were performed by a single surgeon with standardized techniques. To the best of our knowledge, this is one of the largest studies to date evaluating PD surgery with different grafts.

In conclusion, for the surgical treatment of PD, grafting procedures are effective but challenging options. Therefore, they should be applied by experienced centers. It is important to inform patients about the risks of erectile dysfunction and PLL, which may develop in the postoperative period. The ideal graft material has not yet been determined. Porcine pericardial EMGs (XenoGuard), porcine intestinal submucosal EMGs (BioDesign), and bovine pericardial EMGs (Tutopatch) had significantly shorter operative times compared to autologous venous grafts in our practice. Although there was no statistically significant difference, we think that EMGs should be primarily preferred since erectile dysfunction does not develop and LOS has a shorter recovery time. No significant difference was observed between the EMGs. Prospective randomized controlled studies with larger patient groups are needed.

## Figures and Tables

**Table 1:** Demographic features of the patients.

	Group 1	Group 2	Group 3	Group 4	p
Age (Mean ± SD)	52.4 ± 14.4	53.7 ± 10.7	57 ± 8.6	51.6 ± 11.7	0.392
BMI^*^ (Mean ± SD)	25.4 ± 2.5	25.3 ± 2.8	25.7 ± 3.0	24.1 ± 2.3	0.227
Follow-up (Weeks) (Mean ± SD)	80.3 ± 27.8	39.3 ± 11.1	56.1 ± 14.9	62.6 ± 10.9	0.035
PD Duration (months) (Mean ± SD)	15.0 ± 4.7	15.4 ± 5.0	16.0 ± 3.9	16.2 ± 2.3	0.298
HT, n (%)	19 (52.8)	18 (41.9)	18 (45)	13 (36.1)	
DM, n (%)	15 (41.7)	21 (48.8)	13 (32.5)	10 (27.8)	
CHD, n (%)	5 (13.9)	6 (14)	6 (15)	6 (16.7)	
Trauma, n (%)	3 (2.8)	2 (2.3)	5 (12.5)	7 (19.4)	0.058
HL, n (%)	6 (16.7)	8 (32.6)	5 (7.5)	4 (5.6)	0.065
Smoking, n (%)	22 (61.1)	29 (67.4)	23 (57.5)	19 (36.1)	0.092

BMI: Body-mass index, HT: Hypertension, DM: Diabetes mellitus, CHD: Coronary heart disease, HL: Hyperlipidemia, PD: Peyronie’s disease, SD: Standard deviation.

**Table 2:** Preoperative and postoperative IIEF-5 scores, graft size, and operation time (Mean ± SD).

	Group 1	Group 2	Group 3	Group 4	p
Preoperative IIEF-5	16.61 ± 5.484	18.77 ± 4.937	17.6 ± 2.881	17.94 ± 2.317	0.143
Postoperative IIEF-5	17.78 ± 4.065	19.93 ± 4.506	17.85 ± 3.142	19.44 ± 2.157	**0.011**
Graft size (mm^2^)	198.47 ± 160.566	224.26 ± 182.205	154.75 ± 90.44	156.25 ± 74.011	0.460
Surgical duration (min)	134.83 ± 17.907	65.47 ± 6.991	63.08 ± 6.642	62.97 ± 7.117	**<0.001**

IIEF-5: International Index of Erectile Function.

SD: Standard deviation.

**Table 3:** Intraoperative features.

		Group 1 (n, (%))	Group 2 (n, (%))	Group 3 (n, (%))	Group 4 (n, (%))
Curvature degree (≥60°)		24 (66.7)	24 (55.8)	7 (17.5)	8 (22)
Complex deformities		2 (5.6)	2 (4.7)	0 (0.0)	3 (8)
Curvature direction	Dorsal	12 (33.3)	20 (46.5)	19 (47.5)	19 (53)
	Ventral	7 (19.4)	7 (16.3)	5 (12.5)	2 (6)
	Right lateral	3 (8.3)	1 (2.3)	0 (0.0)	4 (11)
	Left lateral	8 (22.2)	3 (7.0)	7 (17.5)	5 (14)
	Dorsal-right lateral	0 (0.0)	2 (4.7)	2 (5.0)	1 (3)
	Dorsal-left lateral	4 (11.1)	7 (16.3)	2 (5.0)	4 (11)
	Ventral-right lateral	1 (2.8)	1 (2.3)	2 (5.0)	1 (3)
	Ventral-left lateral	1 (2.8)	2 (4.7)	3 (7.5)	0 (0)
Cavernous sacculation		7 (19.4)	1 (2.3)	2 (5.0)	1 (3)
Urethra dissection		15 (41.7)	22 (51.2)	7 (17.5)	3 (8)

**Table 4:** Postoperative complications.

		Group 1 (n, (%))	Group 2 (n, (%))	Group 3 (n, (%))	Group 4 (n, (%))
Wound infection	Yes	0 (0.0)	0 (0.0)	0 (0.0)	0 (0.0)
Hematoma	Yes	2 (5.6)	2 (4.7)	0 (0.0)	1 (3)
Penile edema	Yes	8 (22.2)	4 (9.3)	1 (2.5)	0 (0.0)
Incision contracture	Yes	0 (0.0)	0 (0.0)	0 (0.0)	0 (0.0)
Venous leak	Yes	3 (8.3)	3 (6.9)	0 (0.0)	0 (0.0)
Ballooning of the graft	Yes	0 (0.0)	0 (0.0)	0 (0.0)	0 (0.0)
Inguinal hypoesthesia	Yes	0 (0.0)	0 (0.0)	0 (0.0)	0 (0.0)
Anejaculation	Yes	0 (0.0)	0 (0.0)	0 (0.0)	0 (0.0)
Painful plication suture	Yes	0 (0.0)	0 (0.0)	0 (0.0)	0 (0.0)
Palpable suture	Yes	0 (0.0)	0 (0.0)	0 (0.0)	0 (0.0)

**Table 5:** Postoperative loss of sensation and penile length loss by groups.

	Group 1	Group 2	Group 3	Group 4	p
Loss of sensation (days) (Mean **± SD)**	18.94 ± 4.869	14.81 ± 5.901	12.33 ± 5.366	15.17 ± 3.103	**<0.001**
Penile length loss (mm) (Mean **± SD)**	5.83 ± 7.02	5 ± 6.637	2.75 ± 4.929	1.81 ± 4.167	**0.017**

SD: Standard deviation.

**Table 6:** Relationship between penile curvature degree and loss of sensation and penile length loss.

	Curvature degree	Mean ± SD	p
Graft size (mm^2^)	0–59	164.5 ± 102.8	0.234
	≥60	213.7 ± 174.8	
Loss of sensation (days)	0–59	14.4 ± 5	**0.028**
	≥60	16.4 ± 5.8	
Penile length loss (mm)	0–59	2.6 ± 5	**0.002**
	≥60	5.7 ± 6.8	

SD: Standard deviation.
